# Transcriptional analysis of the multiple *Sry* genes and developmental program at the onset of testis differentiation in the rat

**DOI:** 10.1186/s13293-020-00305-8

**Published:** 2020-05-12

**Authors:** Jeremy W. Prokop, Surya B. Chhetri, J. Edward van Veen, Xuqi Chen, Adam C. Underwood, Katie Uhl, Melinda R. Dwinell, Aron M. Geurts, Stephanie M. Correa, Arthur P. Arnold

**Affiliations:** 1 Department of Pediatrics and Human Development, College of Human Medicine, Michigan State University, Grand Rapids, MI 49503 USA; 2 Department of Pharmacology and Toxicology, Michigan State University, East Lansing, MI 48824 USA; 3 HudsonAlpha Institute for Biotechnology, Huntsville, AL 35806 USA; 4 Johns Hopkins University, Baltimore, MD 21218 USA; 5 Department of Integrative Biology & Physiology, Laboratory of Neuroendocrinology of the Brain Research Institute, University of California, 610 Charles Young Drive South, Los Angeles, CA 90095 USA; 6 Division of Mathematics and Science, Walsh University, North Canton, OH 44720 USA; 7 Department of Physiology, Medical College of Wisconsin, Milwaukee, WI 53226 USA

**Keywords:** Sry, Testis determination, Sex determination, *Rattus norvegicus*, RNAseq, Transcriptome, Gonadal ridge, Embryo

## Abstract

**Background:**

The commonly used laboratory rat, *Rattus norvegicus*, is unique in having multiple *Sry* gene copies found on the Y chromosome, with different copies encoding amino acid variations that influence the resulting protein function. It is not clear which *Sry* genes are expressed at the onset of testis differentiation or how their expression correlates with that of other genes in testis-determination pathways.

**Methods:**

Here, two independent E11–E14 developmental RNAseq datasets show that multiple *Sry* genes are expressed at E12–E13.

**Results:**

The identified copies expressed during testis initiation include *Sry4A*, *Sry1*, and *Sry3C*, which are conserved in every strain of *Rattus norvegicus* with genomes sequenced to date.

**Conclusions:**

This work represents a first step in defining the complex environment of rat testis differentiation that can open the door for generating sex reversal model systems using embryo manipulation techniques that have been available in the mouse but not the rat.

## Introduction

Differentiation of the testes in most metatherian and eutherian mammals is initiated by SRY, encoded by the Y chromosome [1–3]. Knockout and transgenic experiments in mice demonstrate that *Sry* is critical for the development of the testes [4]. SRY expression in somatic cells of the undifferentiated gonadal ridge activates a cascade of testis-differentiating genes including *Sox9* [5, 6]. In humans, SRY is expressed also in germ cells [7]. In humans and rodents, SRY is also expressed throughout adulthood in non-gonadal tissues such as the brain and adrenals [8, 9]. In rats, the *Sry* gene has been duplicated through gene conversion at transposable elements on the Y chromosome [10]. Common laboratory strains of rats have at least 11 copies of *Sry* genes (*Sry1*, *Sry2*, *Sry3*, *Sry3A*, *Sry3B*, *Sry3BI*, *Sry3BII*, *Sry3C*, *Sry4*, *Sry4A*, *nonHMG Sry*) [10–12], some of which are expressed in many non-gonadal tissues. Amino acid variants among different *Sry* proteins alter biological function [10, 13]. Two unusual variants include *nonHMG-Sry* and *Sry2*. The former encodes a protein lacking the HMG domain common to all SOX family proteins, required for DNA binding and SOX protein function. The *Sry2* gene is located within the gene coding region of *Kdm5d* and shows ubiquitous expression in male tissues, yet contains a mutation that reduces nuclear localization of the protein (~ 50% retained cytoplasmic localization) and alters transcriptional potential [10]. Analysis of numerous RNAseq datasets shows that diverse *Sry* genes other than *Sry2* are expressed in a pattern overlapping with that of human *SRY* expression [13].

A major unresolved question in laboratory rats is which *Sry* gene(s) cause(s) testis differentiation. One copy may be sufficient, with others being redundant (or not), or several *Sry* copies of varying function may all be required. Here, we sequenced RNA from rat gonads at the time of testis differentiation to determine which *Sry* genes are expressed when the testis-determining factor is active. Three *Sry* genes (*Sry1*, *Sry4A,* and *Sry3C*), other than *Sry2*, were found to be expressed at the highest levels at E13, the time of testis differentiation, a result that narrows down the *Sry* genes that likely act as the testis-differentiating factor. This is the first report of multiple *Sry* genes potentially contributing to testis differentiation in a mammal. The results represent an important step forward in understanding testis differentiation in one of the most highly studied animal model systems used in biomedical research. This information will be essential for understanding the function of the different *Sry* genes in gonadal development and for engineering rat models that genetically separate the effects of sex chromosomes and circulating hormones on sexual differentiation, similar to the Four Core Genotypes mouse model [14, 15].

## Methods

### Animals

Timed pregnant Sprague Dawley CD1 rats were obtained from Charles River (San Diego, CA, USA). In experiment 1, pregnant females were euthanized at embryonic day 11, 12, 13, and 14 (E0 = day of seminal plug). Tissue was collected from the urogenital ridge, including gonadal anlage and adjacent tissue, as described below. This analysis provided a preliminary assessment of gene expression across embryonic ages before and after onset of testis differentiation, with one or two independent pooled samples per age. E11 and 14 fetuses were staged by limb morphology [16]. E12 and E13 fetuses were precisely staged by counting the number of tail somites (ts) [17]. In each E11 fetus, a single urogenital complex was collected from both sides, including both urogenital ridges and both pronephroi (*n* = 2 pools). For the E12 (10–12 ts) fetus (*n* = 1 pool) and E13 (21–22 ts) fetuses (*n* = 2 pools), two urogenital ridges, which include the gonadal ridge and mesonephros, were collected. At E14, two gonads, dissected away from the mesonephroi, were collected and pooled (*n* = 1 pool). In experiment 2, we used the preliminary data from experiment 1 to select one specific age (E13) with the highest expression of *Sry* and dissected only gonadal tissue, with much higher sampling (*n* = 5 independent samples from different fetuses), to focus on expression at the time of putative onset of testis differentiation. All 5 samples were gonad pairs, dissected away from the mesonephroi, from E13 (21 ts) XY fetuses. All samples were sexed by PCR for X-Y copies of *Med14* (primers F: CCTCCAGACCCTATTACCAA, R: ATCACTGTCAAGGTTGCTTC), which produces a 560 bp PCR product in XX females and 560 bp and 172 bp products in XY males.

### RNAseq

For experiment 1 (BioSample SAMN13930124), RNA was extracted from urogenital ridge of E11 and E12, the genital ridge/mesonephros of E13, and testes of E14 fetuses using Trizol (Invitrogen, Carlsbad, CA, USA) and treated with RNase-free DNase (Promega, Madison, USA) to remove possible genomic DNA contamination. RNA quality was checked using an Agilent TapeStation. RNAseq was processed at the HudsonAlpha Institute for Biotechnology (HAIB) Genomic Services Laboratory using PolyA capture and sequenced on Illumina HiSeq v4 generating 50 bp paired end reads (Table 1).

**Table 1:** RNAseq samples for NCBI BioProject PRJNA603367

Name	Day	Experiment number	NCBI SRA accession	Location of RNAseq	# Spots	GC %
E11	E11	1	SRR10971979	HAIB	76,755,285	52.53
E11_2	E11	1	SRR10971978	HAIB	82,683,942	52.70
E12	E12	1	SRR10971976	HAIB	66,920,760	51.84
E13	E13	1	SRR10971975	HAIB	89,604,541	50.80
E13_2	E13	1	SRR10971974	HAIB	67,669,568	50.52
E14	E14	1	SRR10971973	HAIB	67,718,710	52.19
21ts_1	E13	2	SRR10971972	UCLA	26,540,107	43.48
21ts_2	E13	2	SRR10971971	UCLA	23,227,742	43.60
21ts_3	E13	2	SRR10971970	UCLA	27,134,789	44.22
21ts_4	E13	2	SRR10971969	UCLA	25,802,203	43.58
21ts_5	E13	2	SRR10971977	UCLA	26,166,729	43.94

For experiment 2 (BioSample SAMN13930125), sequencing was performed on libraries prepared from previously snap-frozen gonads. RNA was isolated using the column-based Qiagen RNeasy Micro kit. RNA integrity was assayed on an Agilent TapeStation. RNA Integrity number (RIN) values for all samples ranged from 8.5 to 9.2. The Nugen Ovation RNA-Seq system V2 was used for reverse transcription of cDNA, and the Nugen Ovation Ultralow system V2 was used to prepare sequencing-ready libraries. Libraries were quantified using Qubit DNA high-sensitivity reagents, and library quality control was performed on an Agilent TapeStation using a D5000 high-sensitivity DNA tape. Sequencing was performed in a single lane of paired-end (2 × 75) Illumina NextSeq 500 using v2 reagents, and later demultiplexed. Reads for all experiments are available online from the SRA as listed in Table 1 and are found in the NCBI BioProject PRJNA603367.

### Bioinformatics

Each of the RNAseq datasets was quantified using Salmon tools [18] against the *Rattus norvegicus* Ensembl transcript build 96. Sample analysis was performed using Network Analyst [19] with comparisons being made with DESeq2 [20] with cutoff Log2 fold change values of > 2 or < − 2 and an adjusted *p* value < 0.01. Gene networks and Gene Ontology (GO) enrichment were performed using STRING [21], such that we clustered genes elevated at each day that are connected to various biological and molecular pathways. *Sry* mapped reads were extracted using Burrows-Wheeler aligner (BWA) [22] against the *Sry1* gene (AY157669.1). Single nucleotide markers for each of the *Sry* genes were used from our previous publication [13] (Fig 2b). Read depth for each of the bases was extracted from the BWA alignment of all mapped RNAseq datasets in addition to each individual analysis using UGENE [23], followed by averaging the percent of each base at every position. Marker spots for each of the *Sry* genes were averaged to determine the relative percent composition of each *Sry* gene, which was then multiplied by the transcripts mapped per million reads sequenced (TPM) to determine composition of *Sry* at each time point.

## Results

Using RNAseq, in experiment 1, we surveyed gene expression in samples of XY male urogenital ridge across embryonic days 11–14, using low sample coverage at each embryonic day, to give a preliminary assessment of the timing of expression of *Sry* and other genes known to be involved in testis differentiation in other mammalian species. Based on those results, E13 was identified as the time with highest expression of *Sry.* In experiment 2, we performed well-powered analysis of gene expression on E13, only in the testis itself, without contamination of adjacent non-gonadal tissues. Using variant mapping between the different copies of *Sry*, it was then possible to determine the percent of total *Sry* each copy makes up, which was used to normalize total *Sry* mapping into each specific copy.

### Differentially expressed genes

**Fig. 1:**
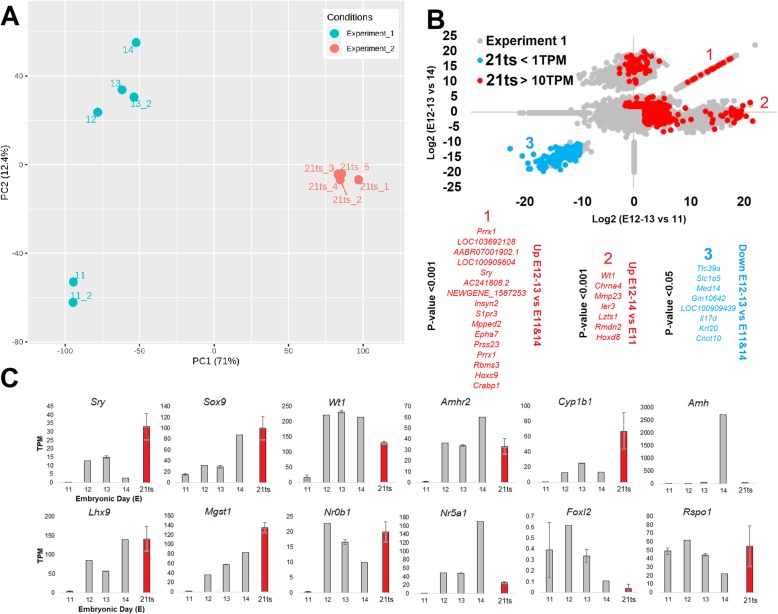
RNAseq of rat developmental time points near male sex determination. **a** Principle components analysis of global gene expression measured by RNAseq done in two experiments (blue = experiment 1 at Hudson Alpha Institute of Biotechnology HAIB, peach = experiment 2 at UCLA). ts, tail somites. **b** Plot of genes in experiment 1 differentially expressed between E12–13 vs E11 (*x*-axis) and E12–13 vs E14 (*y*-axis). Values in blue were for those genes from experiment 1 with differences that in experiment 2 (21 ts, 21 tail somites) had expression values below 1 TPM (transcript per million reads sequenced) and those in red with values > 10 TPM. Three clusters were noted (1–3) with the most significant genes listed in order for each group shown below the chart. **c** Transcripts per million reads sequenced (TPM) for genes known to be involved in testis or ovary differentiation. Experiment 1 values are shown in gray and experiment 2 in red. Error bars represent the standard deviation

The principal components analysis of RNASeq samples shows clustering based on principal component (PC) 1 and 2, with different clustering of E11 and E12/13 samples (experiment 1, Fig. 1a); PC1 stratifying the two different experiments; and PC2 stratifying day of embryonic development. The samples from E13 (21 ts) of experiment 2 cluster differently from those of experiment 1 (Fig. 1a). This is likely due to the difference in tissues sampled in the two experiments. Previous studies have shown testis differentiation begins at E12 in rats [24]. Preliminary gene analysis comparing E11 (*n* = 2) and E12/E13 samples (*n* = 3) from experiment 1 using DESeq2 (adj *p* < 0.01) revealed a significant enrichment, based on gene ontology to the entire genome, for genes involved in gonadal development (12 genes: *Amhr2*, *Bcl2*, *Cga*, *Cyp1b1*, *Inhbb*, *Lhx9*, *Mgst1*, *Nr0b1*, *Nr5a1*, *Ren*, *Sohlh2*, *Wt1*, *FDR* = 0.0015), kidney development (9 genes: *Agtr2*, *Egr1*, *Hrsp12*, *Hspa8*, *Nphs2*, *Ren*, *Sdc4*, *Sulf1*, *Wt1*, *FDR* = 0.023), and core histones (11 genes: *ENSRNOG00000034127*, *Hist1h2aa*, *Hist1h2an*, *Hist1h2bd*, *Hist1h2bf*, *Hist1h2bk*, *Hist1h2bo*, *Hist1h3c*, *Hist2h2ab*, *LOC690131*, *rCG_23123*, FDR = 7.8 × 10^−7^). Because of the differential clustering of E12/13 relative to either E11 or E14, we plotted two comparisons, E12/13 vs E11 (*x*-axis) and E12/13 vs E14 (*y*-axis), identifying multiple genes that, like *Sry*, have increased expression on E12/13 relative to either E11 or E14 (Fig. 1b, *p* < 0.001, group 1); genes that are higher in all days except E11 (Fig. 1b, *p* < 0.001, group 2); and genes that are lower in E12/13 vs. either E11 or E14 (Fig. 1b, *p* < 0.05, group 3). Although the measurement of these genes is preliminary based on the low sample size, there were numerous genes known to be activated in other studies at testis differentiation that are observed with the expected time course in Fig. 1c, suggesting that E13 was the optimal age for further analysis with a higher sample size in experiment 2. The absolute values of gene expression in experiment 1 and several of the genes potentially elevated at E12/13 are subject to replication in future studies because of the low sample size at each age.

The measurement of gene expression exclusively from gonadal tissue at E13 (21 ts) in experiment 2 suggests that many genes unique to E12/13 groups are from the genital ridge and not from the mesonephros (Fig. 1b, colored dots). The expression of several markers for sexual differentiation of the gonads at each of the days of rat development fall within these groups that begin to increase in expression at E12 of experiment 1 (*Sry*, *Sox9*, *Wt1*, *Amhr2*, *Cyp1b1*, *Lhx9*, *Mgst1*, *Nr0b1*, *Nr5a1*; Fig. 1c), whereas experiment 2 (21 ts) further confirms the presence of these markers. *Amh* increased expression later, at E14. Ovary markers *Foxl2* and *Rspo1* appeared to be downregulated by E14 (Fig. 1c). Our bulk dissection of developmental days (experiment 1) confirms that E13 corresponds to the beginning of testis determination. Fine dissections of 21 ts embryos with an *n* of 5 validate the presence of *Sry*, allowing for repeated independent assessments of *Sry* copies at testis differentiation.

### Expression of different Sry genes

**Fig. 2:**
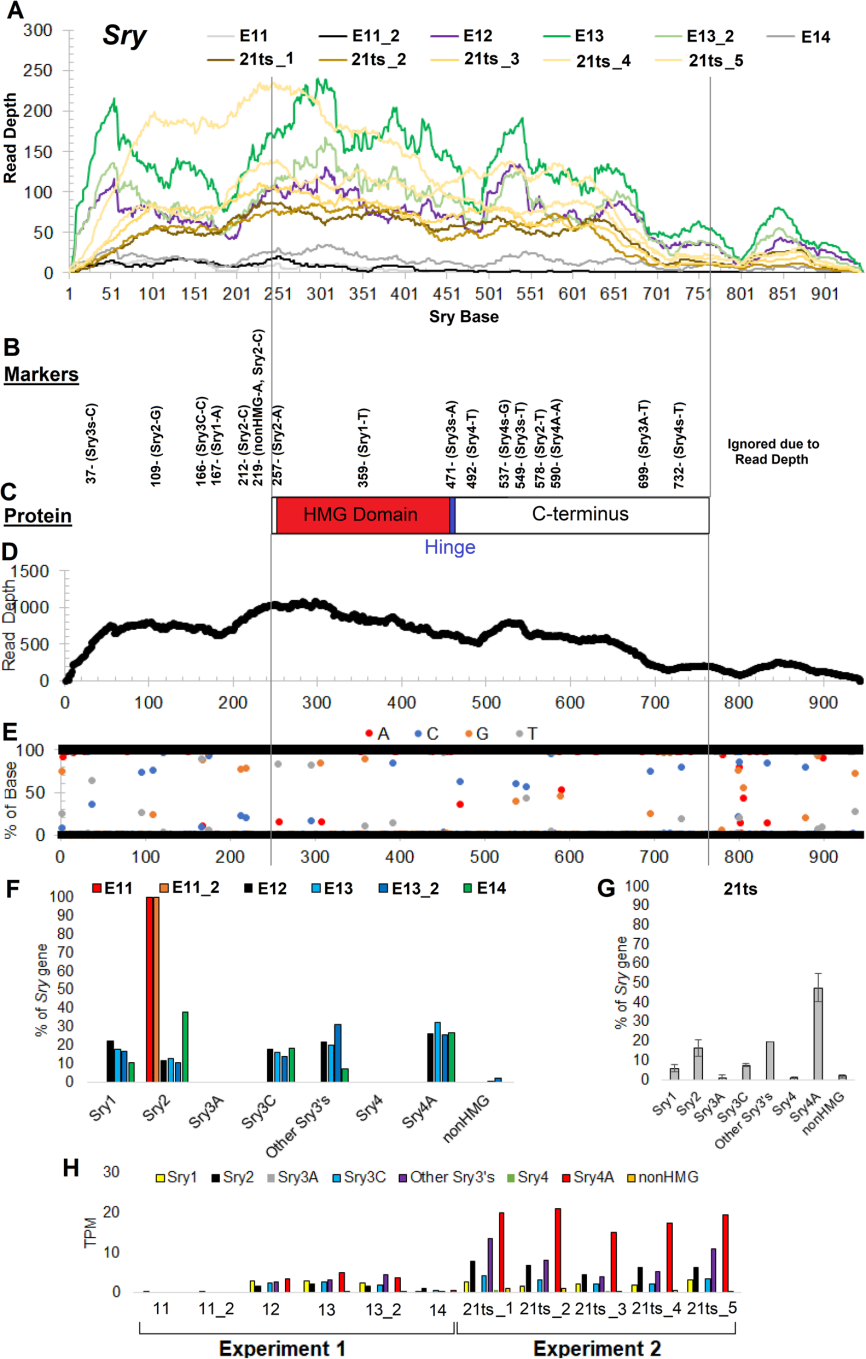
*Sry* gene mapping in RNAseq. **a**–**e** All panels of *Sry* mapped data are aligned relative to the transcript positions with the coding region marked by gray lines. **a** Depth of reads aligned to *Sry* for each sample representing different embryonic stages. **b** Single nucleotide variants along the *Sry* genes used for determining *Sry* copies expressed. **c** SRY protein corresponding to regions of the gene aligned to panels **a** and **b**. **d** Total read mapping over the *Sry* gene from all datasets. **e** The percent of each base (*y*-axis) at positions along the *Sry* gene (*x*-axis). Bases with values between 0 and 100 are those with variants in the different *Sry* genes seen in reads generated from all experiments. Values of 0 and 100 are shown as black line with any positions outside of these shown. Bases are listed as A = red, C = blue, G = orange, and T = gray. **f**, **g** The percent of each *Sry* gene using markers from panel **b** for the various days of experiment 1 (**f**) and analysis of multiple E12 (21 ts, tail somites) embryos in experiment 2 (**g**) with standard deviation shown as error bars. The high percentage expression of *Sry2* at E11, despite its low abundance (**h**), results from the absence of expression of other *Sry* genes at E11. **h** Conversion of total *Sry* TPM using percent of each *Sry* gene to determine the individual *Sry* gene TPMs. In experiment 1, two samples were measured on E11 and 13. In experiment 2, 5 samples were measured from fetuses with 21 tail somites

Expression of *Sry* increased at E12 and E13, correlated with initiation of testis differentiation, suggesting that these RNAseq runs have the power to segregate copies of different *Sry* genes by sequence during this period. We began by measuring the read depth for *Sry* in each of the RNAseq files (Fig. 2a). A total of 16 variants were identified to differentiate the *Sry* genes (Fig. 2b), 10 of which were found within the protein-coding region (Fig. 2c). Compiling all reads of *Sry* genes in all RNAseq runs, the average depth at any site within the gene is 563 reads, with > 150 read coverage at amino acids 9-779 (Fig. 2d), where 1 is the first base of the transcript. Using the compiled data, 25 locations have variation of bases within the reads, with all of our markers showing variation (Fig. 2e) and high coverage (197–1007 reads). We removed markers that fall below 150 read depth, removing many variants that show up on the 3′ end of the transcript. With the markers, we calculated the percent of *Sry* reads that map to each of the genes. At E11 (experiment 1), prior to testis differentiation, the ubiquitously expressed *Sry2* represented 100% of reads (Fig. 2f), even though absolute numbers of reads was low at this time point (Fig. 2a gray and black). Over the period E12–E14 (experiment 1), we identified *Sry1*, *Sry3C*, *Sry4A*, and “other *Sry3s*” (transcripts for which reads did not allow further assignment to specific *Sry3* genes) (Fig. 2f). Analysis of data from E13 in experiment 2 confirmed that *Sry4A* was expressed at the highest level, together with other *Sry3s*, *Sry2*, *Sry3C*, and *Sry1* (Fig. 2g). Very low levels of expression on all days were found for *Sry3A*, *Sry4*, and the *nonHMG Sry*, suggesting these genes have minimal involvement in testis differentiation. Normalizing the globally mapped TPM values on each RNAseq dataset for *Sry* using the percent composition of each copy shows a relatively high value of *Sry4A*, other *Sry3s*, *Sry3C*, and *Sry1* starting at E12, continuing at E13, and decreasing at E14 (Fig. 2h).

## Discussion

The present findings suggest that *Sry* genes are expressed in a narrow window at the onset of rat testis development, beginning at E12/E13 and declining on E14. *Sry4A* may not decrease in expression by E14. This result supports the idea that E12 is the first day of testis differentiation in Sprague Dawley rats. The onset of *Sry* expression appears to mirror that previously shown in mice and rats and supports the idea that *Sry* expression in rat gonadal ridge may not be needed for testis differentiation after its brief peak on E12/E13. Because Sry2 is expressed in testis before and after E12/13 and in nearly all male non-gonadal tissues in adult [13], we discount its possible role in causing testis differentiation, especially because it lacks an efficient nuclear translocation signal. The results further support the idea that the top candidate testis-differentiating gene is *Sry4A*, because it is expressed at E13 higher than any other single *Sry* gene, and in some individuals higher than all other *Sry* genes combined (Fig. 2h). *Sry1* and *Sry3C* are also expressed at significant levels. Thus, *Sry4A*, *Sry3C*, and *Sry1* are the top candidates for transgenic manipulation to cause sex reversal. These results suggest that *Sry3A*, *Sry4*, and the *nonHMG Sry* are not significantly expressed and therefore are not involved in testis differentiation. The data do not discriminate between *Sry* expression in the Sertoli cell somatic lineage vs. germline cells and do not resolve if several *Sry* genes are required or redundant for the testis-differentiation cascade. SRY expression is found in human germ cells [7] and in rat testis [13]. Our previous work suggests that Sry3s are the primary transcripts in adult rat testis, and here, we suggest that Sry4A is expressed more at the time or testis differentiation.

The *Sry* genes in rodents are known to differ from human *SRY* in that they lack the N-terminal region that contains phosphorylation signal sites suggested to be involved in *SRY* activation [25]. Instead, the rodent *Sry* genes have an extended C-terminal polyglutamine tract that is essential for testis differentiation and required for the activation of *Sox9* [26]. This region contains a high concentration of CAG coding glutamine codons with an additional high level of “FHDH” motifs. Interestingly, the rat *Sry* genes (~ 25 amino acids) have a shorter polyglutamine tract than mouse (240 amino acids), with the different *Sry* genes having variation in the length and compositions of the tract [10] suggesting continued drift of the region following duplication of genes. This unstable CAG region has allowed for rapid evolution of *Sry* genes in rodents, with suggestions that the length of the region determines *SRY* activation potential [27]. Thus, mouse *Sry* has a higher activation potential with the larger polyglutamine tract than rat *Sry*. Our data presented here suggest the interesting hypothesis that unlike human with the activation potential of the N-terminus or the mouse long polyglutamine tract, rat has evolved the use of multiple copies of the *Sry* genes to reach activation levels required to initiate testis differentiation. Further studies are required to test this hypothesis.

This RNAseq work also extends our group’s previous mapping all possible *Sry* copies found on the rat Y-chromosome. Here, we have added studies of the Sprague Dawley strain, but find no newly identified variants that would suggest unmapped copies in this strain. Our previous work has identified *Sry1*, *Sry4A*, and *Sry3C*, reported here as potential genes causing testis differentiation, to be present in the SHR, WKY, FHH, FHL, ACI, SR, SS, and F344 rat strains [13], further supporting the conservation of these copies in nearly all common rat strains used in research. From the sequenced SHR Y-chromosome of rat [12], we know that all but the *Sry2* copy of *Sry* genes in rat have several thousand base pairs of shared sequence 5′ and 3′ to the gene [10]. From amino acid comparisons, we also know that *Sry1* most resembles human and mouse *Sry*, *Sry4A* contains a variant L98V that has little alteration of SRY transcriptional function [10], and *Sry3C* contains a P76T variant that slightly impacts the protein’s transcriptional potential [28]. However, all of these copies have been shown to be functional SRY proteins, unlike the ubiquitously expressed *Sry2* copy that has variants H4Q and R21H known to impact protein nuclear localization and a shortening of the polyglutamine tract that decreases activation [10]. The identification of *Sry2* in E11 confirms our analysis of *Sry* in RNAseq that identified *Sry2* in all analyzed male samples [13], suggesting a ubiquitous expression that is in line with being unable to initiate testis differentiation.

## Conclusion

Our findings agree with previous evidence that *Sry* genes other than *Sry2* regulate testis differentiation, and identify *Sry1*, *Sry4A*, and *Sry3C* as top candidates for the testis-differentiating gene(s). All of these are conserved in all common laboratory rat strains.

## Data Availability

All RNAseq data can be obtained from NCBI under BioProject PRJNA603367. The SRA-deposited RNAseq FASTQ files can be obtained as accession codes (each of paired end reads available) SRR10971979, SRR10971978, SRR10971976, SRR10971975, SRR10971974, SRR10971973, SRR10971972, SRR10971971, SRR10971970, SRR10971969, and SRR10971977.
